# Orientation of tree rows in alley cropping systems matters – The “ShadOT” modelling tool for tree growth and shading effects

**DOI:** 10.1016/j.mex.2023.102282

**Published:** 2023-07-07

**Authors:** Marco Donat, Jonas Geistert, Kathrin Grahmann, Sonoko D. Bellingrath-Kimura

**Affiliations:** a Leibniz Centre for Agricultural Landscape Research, 15374, Müncheberg, Germany; b Department of Agronomy and Crop Science, Humboldt-University of Berlin, 14195, Berlin, Germany

**Keywords:** Blender 3D, Transition zones, Solar radiation, Agroforestry, High value trees, Sorbus torminalis, “ShadOT” – A modelling tool for tree growth and shading effects in alley cropping systems

## Abstract

Agroforestry systems have received a significant attention in recent years and can be considered as a potential strategy in agricultural production to respond to worsening climatic conditions. The decision-making process for farmers to design and implement agroforestry systems is complex due to time-consuming processes of planting, growing and management of trees, as well as the long-term impacts on the field and its productivity. The shading of the arable land by trees is a core issue and should be reduced through a north-south orientation of the tree rows. However, this orientation is often in conflict with other criteria. In order to consider future shading from different tree row orientations into the design process, the modelling tool “ShadOT” was developed. This tool can simulate tree growth and analyses spatial shading over variable time periods by using only a limited number of parameters. This tool was programmed exclusively with open source software and can therefore be easily extended. It offers an ideal platform for testing different agroforestry designs due to its simple approach and minimal parameterization. Two different designs (north-south and west-east orientation) were tested for a field and differences in the temporal and spatial distribution of shaded areas are presented.

• Modelling tool for tree growth and shading effects is presented.
• The tool is written in Python programming language, uses only open-source software and requires a limited number of inputs.
• Identification of spatial-temporal shading patterns of different alley cropping scenarios.

## Graphical abstract

**Figure:**
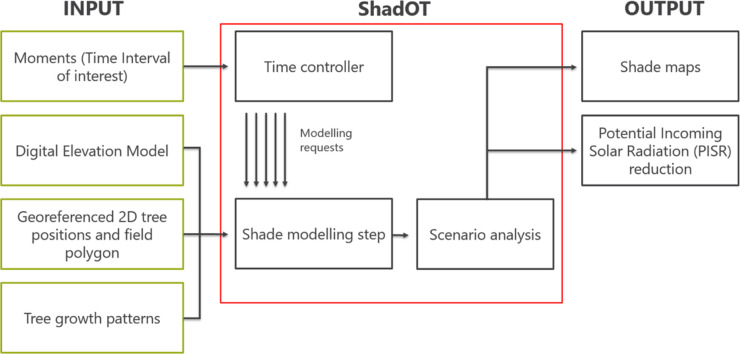

Specifications table

**Table:** 

Subject area:	Agricultural and Biological Sciences
More specific subject area:	Agroforestry modelling
Name of your method:	“ShadOT” – A modelling tool for tree growth and shading effects in alley cropping systems
Name and reference of original method:	N.A.
Resource availability:	Blender—A 3D Modelling and Rendering Package, Stichting Blender Foundation, Amsterdam. Community, B. [8]. System for automated geoscientific analyses (SAGA) v. 2.1. 4. Geoscientific Model Development 8. Conrad, O., Bechtel, B., Bock, M., Dietrich, H., Fischer, E., Gerlitz, L., Wehberg, J., Wichmann, V., and Böhner, J. (2015).

## Background

As a way to address global challenges such as climate change, loss of biodiversity, and environmental degradation, agroforestry is gaining broader acceptance as a potential approach to improve ecosystem services in sustainable intensified cropping systems [31]. When planning and establishing semi-natural habitats e.g. alley cropping, hedgerow or groups of trees into croplands, the possible consequences on the neighbouring agricultural areas (transition zones) must be taken into account. Effects like shading and reduction of wind speeds can lead to microclimatic changes in the transition zones due to different processes such as interception, evaporation and transpiration [15]. Depending on the cardinal orientation of the aforementioned habitats, areas with varying degrees of shading may occur in the transition zones because of the daily and annual path of the sun. The agronomical impact of the light regime is unquestionable, resulting in potential yield losses close to tree rows. For example, on a regional level in Brandenburg, Germany, the yield reduction on maize and wheat from shading at forest-field transition zones amounted 5% to 8% in simulations of a crop model [23]. In Belgium, wheat yield losses even amounted 20% due to an artificial periodic shading experiment [1]. For the maximum reduction of shading effects, a north-south orientation of tree rows is recommended for temperate systems, since the shadow of the tree rows falls into its own row in the most radiation-intensive noon time [4]. If a different spatial orientation is used for the tree rows instead of the north-south direction, additional shading can have adverse effects such as delayed crop development [28], increased relative humidity and hence susceptibility to fungal diseases [6] and reduced yield or crop quality [22]. However, shading agricultural areas reduces evapotranspiration (evaporation of soil water) and therefore improves water supply in dry periods [24], and thus prevent heat damage to plants especially on arable sites with low yield potential [26]. This could increase yield stability during years with longer drought periods and hence constitutes a possible adaption strategy to climate change [15].

Decision criteria such as the main wind direction and terrain slope should be taken into account when implementing habitat structures in order to prevent possible wind [32] and water erosion events [17]. For an optimum protection against wind erosion, the spatial orientation of the habitat strip should be chosen at a 90° angle to the primary wind direction [32]. This orientation in space often deviates from the recommended north-south direction, resulting in additional shaded areas which can lead to a trade-off when arranging alley cropping systems, where criteria for an optimal orientation apparently contradict themselves.

Another important reason to deviate from the north-south orientation is the geometric shape of the field where the agroforestry system will be implemented. For optimal management of crops between rows of trees in an alley cropping system, the number of turning maneuvers for the tractor should be as low as possible to minimize labour time requirements and soil compaction due to increased headland [3,16].

Shading effects in alley cropping systems in particular have received little attention to date due to the challenging task of long-term data collection in established agroforestry systems. Burner et al. [7] investigated the influence of different tree row orientations in a 14-year old alley plantation (*Pinus taeda* L) on alley crop illumination. They used the tree height as input for their SketchUp simulation. Bohn Reckziegel et al. [5] developed a vector based 3D model for real trees after being scanned with a terrestrial laser scanner. Dupraz et al. [10] have developed a complex agroforestry model that considers the light interception of the trees. Swieter et al. [29] calculated shading for an existing north-south orientated short rotation alley cropping system by manipulating the digital elevation model for two vegetation periods. They assumed a constant elevation of the strip and quantified the relative solar radiation using the 'Area solar radiation' function in Esri ArcMap. Analysing the effects of transition zones on yield, [23] generated a virtual block (30 × 30 x 20 m height) within a surrounding flat area of 1.2 ha and thus examined shading. Altogether, these models are either highly complex and require excessive data input, do not consider tree growth to estimate future effects, only analyse the shading of individual trees, or do not consider topography or field geometry.

Jacobs et al. [15] pointed towards the lack of studies on alley cropping systems reviewing the effects on microclimate in temperate alley cropping systems. The authors suggested that future studies should focus on the influence of site characteristics (e.g. topography) when investigating the impact of the alley cropping systems’ microclimate. At the same time, the spatial design should be optimized to increase the productivity of the system with simultaneous beneficial effects on the microclimate. This demand for research is of particular importance as the new EU's common agricultural policy (CAP) 2023 – 2027 ensures the legally secure implementation and management as well as the eligibility of alley cropping systems and a great interest in this cropping system will arise amongst farmers [19].

The orientation of tree rows within the field is one of the most crucial aspects of design and implementation, as it affects efficient field management (cultivation direction and therefore number of turns and headland), wind and water erosion, as well as shading, and thus persisting and influencing over decades the fields’ productivity.

The aim of this study is to present a simple tool called “ShadOT” that quantifies the area in a field that will be shaded by growing trees in the future. We consider the topography of the site, position of the trees and tree growth patterns and show results of two scenarios for possible implementation of a temperate high value timbre alley cropping system. With this tool, the difference of shaded areas within the field can be quantified depending on the orientation of the tree rows and hereby supports a validated decision regarding the design of an alley cropping system.

## Method details

### Software

Implementation of shadow modelling was done using the Python programming language (Version 3.8.5), the Blender 3D software [8] and the Blender GIS-addon application for georeferenced data upload. Data handling and analyses was performed using Python API for Blender, Anaconda Distribution (Anaconda Navigator 1.10.0) for package management and Jupyter Notebook (Version 6.1.4) as an interactive development environment.

### Model overview

Pseudocode of ShadOT's ‘Shade modelling step’

**Fig. 1:**
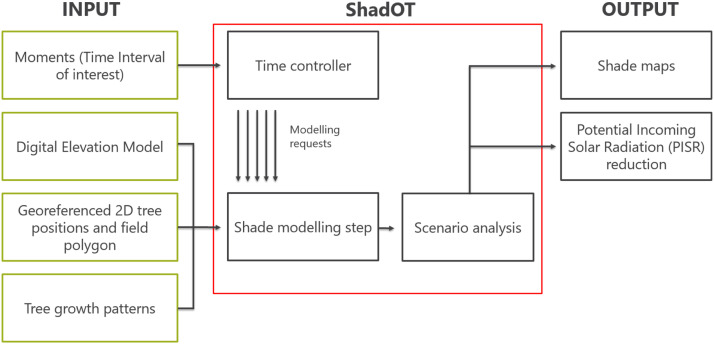
Overview of the functionality of ShadOT. Green boxes on the left side show the input data needed for ShadOT operation. In the red boxes, the elementary components of ShadOT are visualized. The main algorithm “shade modelling step” is described in detail in the Pseudocode section. The time controller provides the required time intervals (e.g. 60 years in 20 min-intervals) and initiates a modelling request. Output of ShadOT are georeferenced shadow maps and Potential Incoming Solar Radiation (PISR) reduction analyses.

The algorithm in the 'Shade modelling step' (see Fig. 1) is presented here as pseudocode (Algorithm 1). This process is performed for every single moment and stored as 'PISR_TreeShade'.

**Algorithm 1:** Shade Modelling Step.

1: FUNCTION shadOT: 2: INPUT: DigitalElevationModel -> dem 3: INPUT: 2DTreePositions -> tpos2d 4: INPUT: TreeGrowthPattern -> tgp 5: INPUT: Moment -> moment 6: 7: CONVERT dem TO 3DTriangleMesh -> surface #[1] 8: CONVERT tpos2d TO 3DTreePosition on surface -> tpos3d #[2] 9: 10: 11: CALC PISR FROM moment,dem -> pisr #[3] 12: CALC SunAltitude,SunAzimut FROM moment-> alti,azi #[4] 13: CALC 3DSunDirectionVector FROM alti,azi -> ray #[5] 14: CALC TreeOcupacity FROM moment,tgp -> treeOcupacity 15: IF alti LESSTHEN 0: 16: RETURN pisr 17: CREATE 3DTreeMesh WITH moment, tgp, tpos3d -> trees3d 18: 19: FOREACH p IN Points FROM surface: 20: IF ray FROM p INTERSECTS trees3d: #[2] 21: # Point in Treeshade 22: PISR_TreeShade[p] = pisr[p] * treeOcupacity 23: ELSE: 24: # Point not in Treeshade 25: PISR_TreeShade[p] = pisr[*p*] 26: 27: RETURN PISR_TreeShade #[1] See BlenderGIS-addon[8] #[2] See Blender Python API*ray_cast*[8] #[3] See SAGA Python API*ta_lighting_2*[9] #[4] See Python Package*Pysolar*[27] #[5] See chapter Sun position

### Case study site

**Fig. 2:**
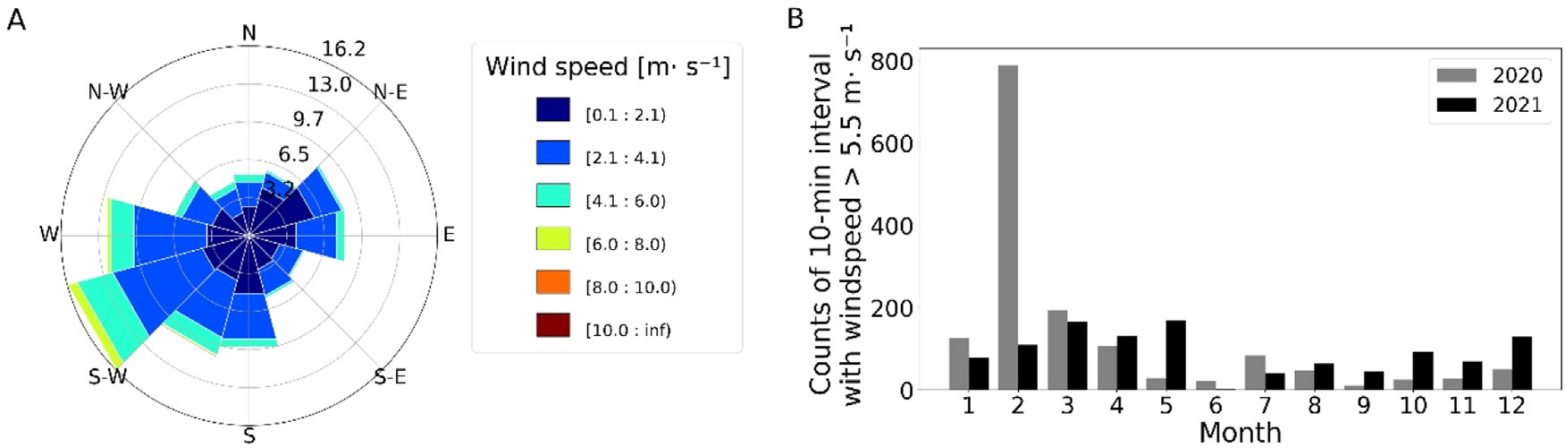
Properties of wind direction and wind speed at DWD Station Neuruppin with a distance of 19 km to the experimental field. A) Wind rose for wind direction in 2020 B) Duration of winds with Beaufort 4 and greater (>5.5 m $s^{-1}$) per month in 2020 and 2021.

The experimental field for model validation was a 6.2 ha agricultural field in a hummocky landscape in northern Brandenburg, Germany. The soil consists of glacial sediments with soil types of loamy and slightly loamy sand and sands. The site is located in the temperate, continental climate zone with a long-term average annual temperature of 9.5 °C with an average annual precipitation of 582 mm [11,12]. The average annual wind speed is 2.4 m $s^{-1}$ in 2020 and 2.3 m $s^{-1}\;$in 2021 with a main wind direction of Southwest (S-W) (Fig. 2A). Months with a large number of 10-minute intervals with high wind speed were recorded in winter and spring months (Fig. 2B). The experimental field has a direct normal irradiation of 967.4$\;kWh\;m^{-2}$ $a^{-1}$, global horizontal irradiation of 1059.9 $kWh\;m^{-2}$ $a^{-1}$, a diffuse horizontal irradiation 558.9 $kWh\;m^{-2}$ $a^{-1}$ and a global tilted irradiation at optimum angle of 1262.1 $kWh\;m^{-2}$ $a^{-1}$ [2].

**Fig. 3:**
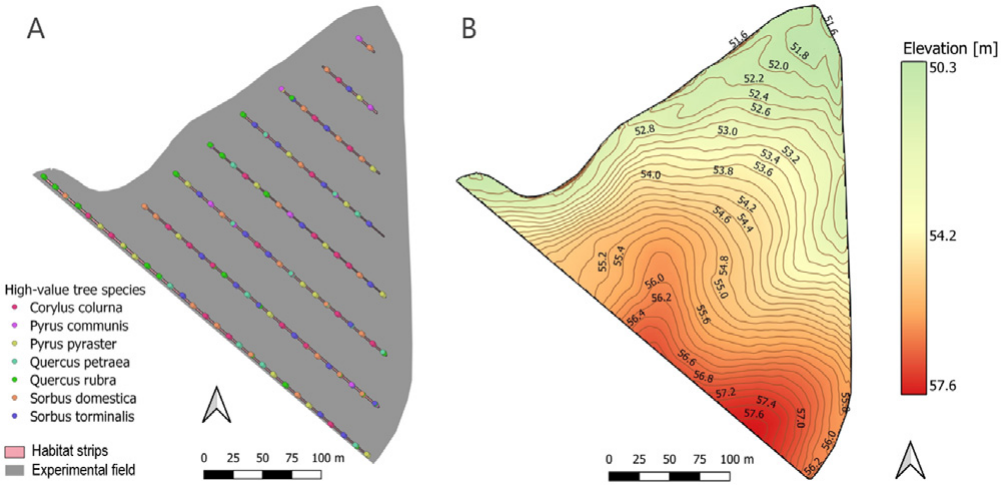
Geospatial layout of the experimental field. A) Design of the alley cropping system implemented in 2017 with the 7 different high value timbre species. B) Topography of the experimental field.

In 2017, 360 individual trees and a total of seven different high-value tree species were planted in groups of three (species of the individual trees within one group was always identical) in the experimental field (Fig. 3A). A distance of 36 m was maintained between eight 2 m wide strips. The habitat strips have a total length of 1.4 km. The distance in the row between tree groups is about 13 m. The site has an average elevation of 54 m above sea level and a total elevation difference of 7.3 m. The site has a gentle slope with a smaller depression in the north (Fig. 3B). The field is conventionally cultivated with a maximum working width of 18 m. The crop rotation from 2017 to 2022 consisted of winter rye, summer oats, winter barley, winter triticale, winter triticale and winter oilseed rape.

### Model scenarios

**Fig. 4:**
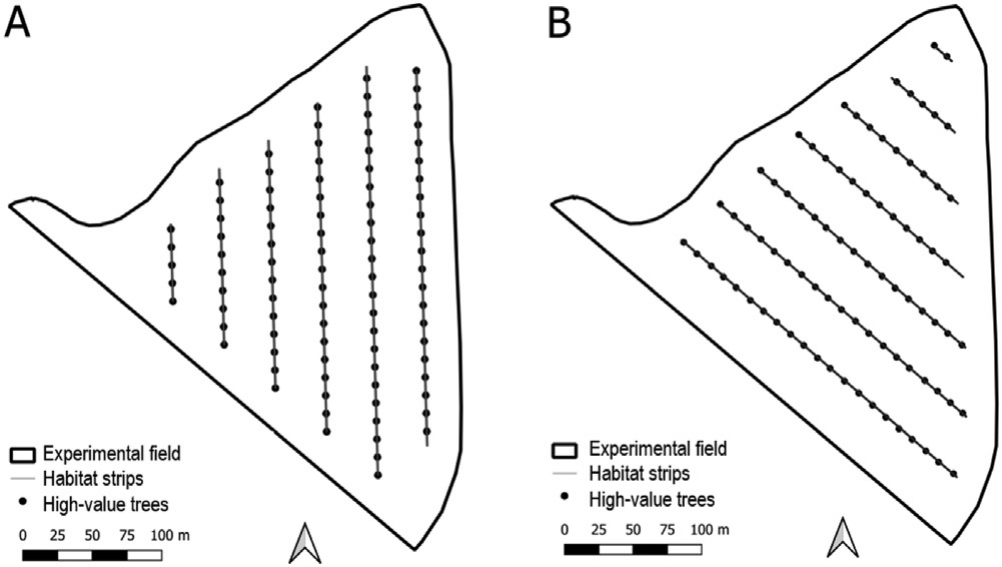
Different Alley Crop designs for the experimental field. A) North-South Scenario B) West-East Scenario - Implemented design at the experimental field with maximal windbreak effect.

In order to test different tree row orientations, ShadOT focused on a single high-value tree species in the test field (wild service tree (Sorbus torminalis (L.) Crantz)). This work is intended to remain close to reality and adheres to the requirements of the new CAP Direct Payments Regulation, according to which agroforestry is only eligible if a minimum distance of 20 m is maintained between the alley and the edge of the field. Therefore, only trees in the inner area of the field were considered and a buffer of 20 m was applied to the field polygon. Two different scenarios (Fig. 4) were compared for ShadOT. One scenario had a north-south orientation of the tree rows with a tree row width of one metre and 32 m spacing between the rows. The spacing between trees within the tree row was 12 m. This resulted in 1163 m of total row length and a total of 92 trees (Fig. 4A). The north-south orientation was chosen for an optimization on shadow reduction. The other scenario had a west-east orientation of the tree rows with a tree row width of one metre and 36 m spacing between the rows. Within the tree row, spacing between individual trees was 12 m. This resulted in 1048 m of total row length and a total of 84 trees (Fig. 4B). The west-east orientation was chosen for an optimization on windbreak effect.

### Moments

ShadOT requires a total time and the length of individual time intervals. These two parameters result in a list of all possible moments that are used as input in the 'Shade modelling step'. For the evaluation of the two scenarios, a total time of 60 years in 20-minute time intervals was used.

### Tree growth patterns

**Fig. 5:**
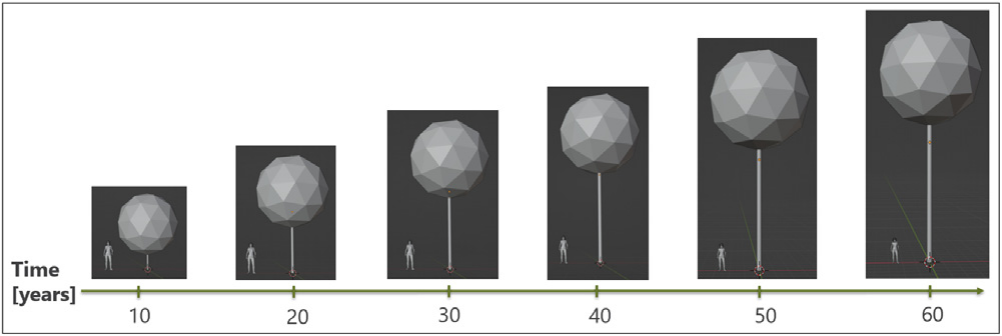
3D Blender visualization of a single tree and the described tree growth patterns over time. To visualize the height and proportions, a person (1.70 m) was placed on the left hand side of the tree.

For both scenarios, tree growth patterns for the tree species wild service tree were used. In the literature, different estimates of the annual height increase, diameter growth and crown diameter were reported [13,20,21,25,30]. In ShadOT, we assumed a constant growth rates of 0.25 m per year. To calculate the crown diameter, a constant diameter growth of 5 mm per year was assumed. To calculate the crown diameter [m], the following formula was used [20]:

(1) Crowndiameter=0.1393x+2.6937

with x being the diameter in breast height. The tree crown was modelled as a sphere. In Fig. 5, the described tree growth patterns as well as the crown shape and proportions over time were visualized using a single tree as an example. For deciduous trees, two different crown opacities were assumed for one calendar year. In the leafless state (November to April) the trees have a crown opacity of 20%, in the foliated state (May to October) it is assumed 75% [14].

### Sun position

**Fig. 6:**
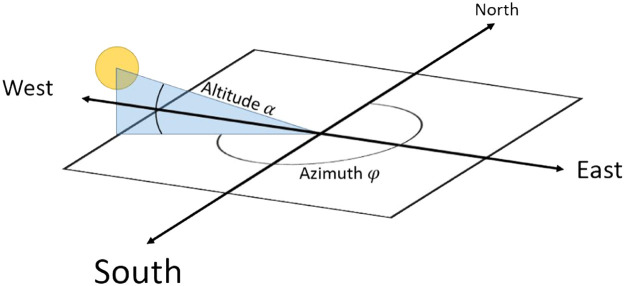
Illustration of Azimuth and Altitude to calculate the position of the sun (changed after [27]).

For the modelling of the shadow, the exact position of the sun was determined for each moment in the period under investigation. Positions of the sun are given in azimuth $\varphi_{deg}$ [°] and altitude $\alpha_{deg}$ [°] (Fig. 6). For all positions of the sun, the module *pysolar.solar* (Python library) was used [27].

Since only solar altitudes above the horizon are relevant, only time intervals with altitude >0° were considered for this study. For the calculation of the position of the sun, it had to be converted into a vector. For this, the angle is needed in radians, which were calculated with the following formulas:

(2) $$\varphi_{rad}=\;\varphi_{deg}\;\pi \;180{{}^{\circ }}^{\;-1}$$

(3) $$\alpha_{rad}=\alpha_{deg}\;\pi \;180^{\circ }{\;}^{-1}$$

Subsequently, the vector was calculated with the following formula:

(4) $$V_{\text{sun}\;}=\left(\begin{array}{c} \cos\left(\alpha_{rad}\right)\sin\left(\varphi_{rad}\right)\\ \cos\left(\alpha_{rad}\right)\cos\left(\varphi_{rad}\right)\\ \sin\left(\alpha_{rad}\right) \end{array}\right)$$

### Digital elevation model

The digital elevation model (DEM) is a crucial data source to understand the topography of a surface and how it will affect the amount of potential solar radiation received at a particular location. Compared to modelling on a flat surface, the modelling of tree shades based on the DEM offers more precise results and a better understanding of the shadow patterns on that particular surface. In this study, the DEM of the experimental field had a resolution of 1 m [18].

### Potential incoming solar radiation

In order to provide additional quantifiable information about the shading events of both scenarios, the Potential Incoming Solar Radiation (PISR) for the field was calculated using the program 'System for automated geoscientific analyses' *SAGA* [9] (SAGA Python API ta_lighting_2).

### PISR reduction

To investigate shaded areas, both scenarios were compared with the sole cropped field without trees. In the sole cropped field, the respective habitat strip (depending on the scenario with which the comparison was made) was also removed, as otherwise differences in the total area would occur. Reduction of PISR was calculated using the following equation:

(5) $$\;\text{PISR}\;\text{Reduction}\;\left[\%\right]\;=\left(1-\frac{PISR-scenario}{PISR-No\;trees}\right)100\%$$

**Fig. 7:**
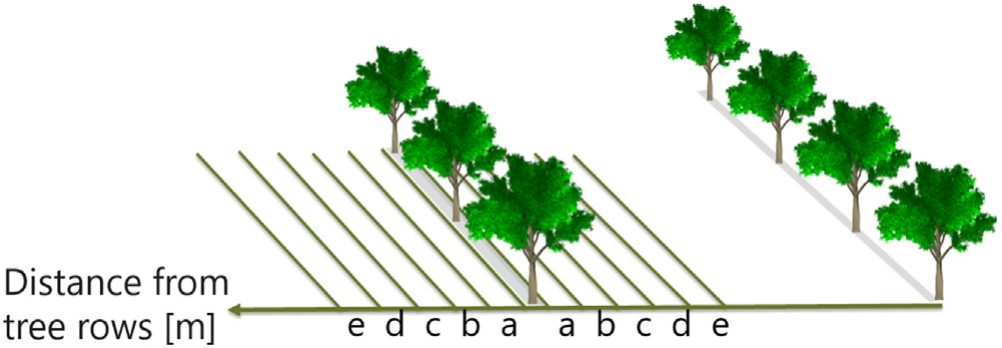
Visualization of different distance classes for the analysis of the shading effects on both sides of the tree rows.

To perform spatio-temporal analyses, PISR was investigated in different distance classes defined as length interval (Fig. 7). The distance classes were created for both sides of all tree rows with the distances of a) 0.5 m to 3 m, b) 3 m to 6 m, c) 6 m to 9 m, d) 9 m to 12 m and e) 12 m to 15 m.

### Scenario analyses

**Fig. 8:**
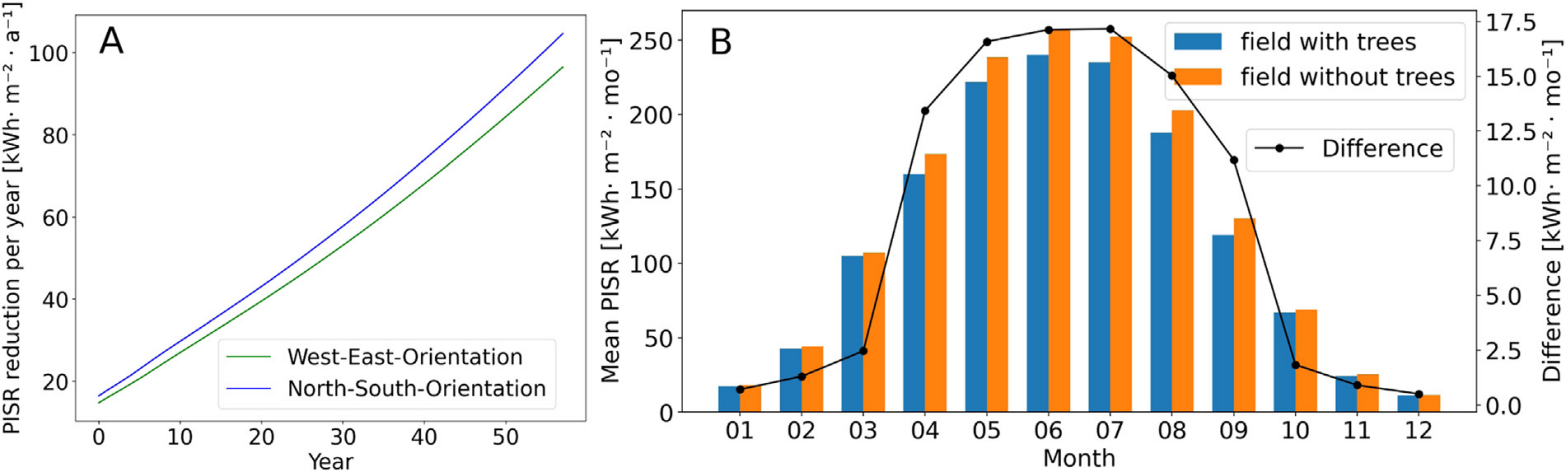
A) Mean PISR reduction per year for the entire field and both scenarios. B) Comparison between mean PISR per month for the entire field (West-East Scenario and year 60) and the field without trees.

In total, there were 13,375 relevant 20-minutes intervals per year for the test field. This results in a total of 802′5000 outputs per scenario, modelling tree growth over 60 years. With increasing age of the trees, the total PISR of the field decreases in both alley cropping scenarios. The mean reduction in PISR per m^2^ was greater in the north-south orientation than in the west-east orientation (Fig. 8). This could be explained by the higher number of trees in the north-south orientation (8 trees more). 60 years after planting, a mean PISR of 1432.2 $kWh\;m^{-2}$ $a^{-1}$ was observed for the field and the west-east scenario, and 1323.9 $kWh\;m^{-2}$ $a^{-1}$for the north-south scenario. In total, this corresponds to a reduction of 6.03 GWh per year for the west-east scenario and 6.53 GWh per year for the north-south scenario for the entire field in year 60.

**Fig. 9:**
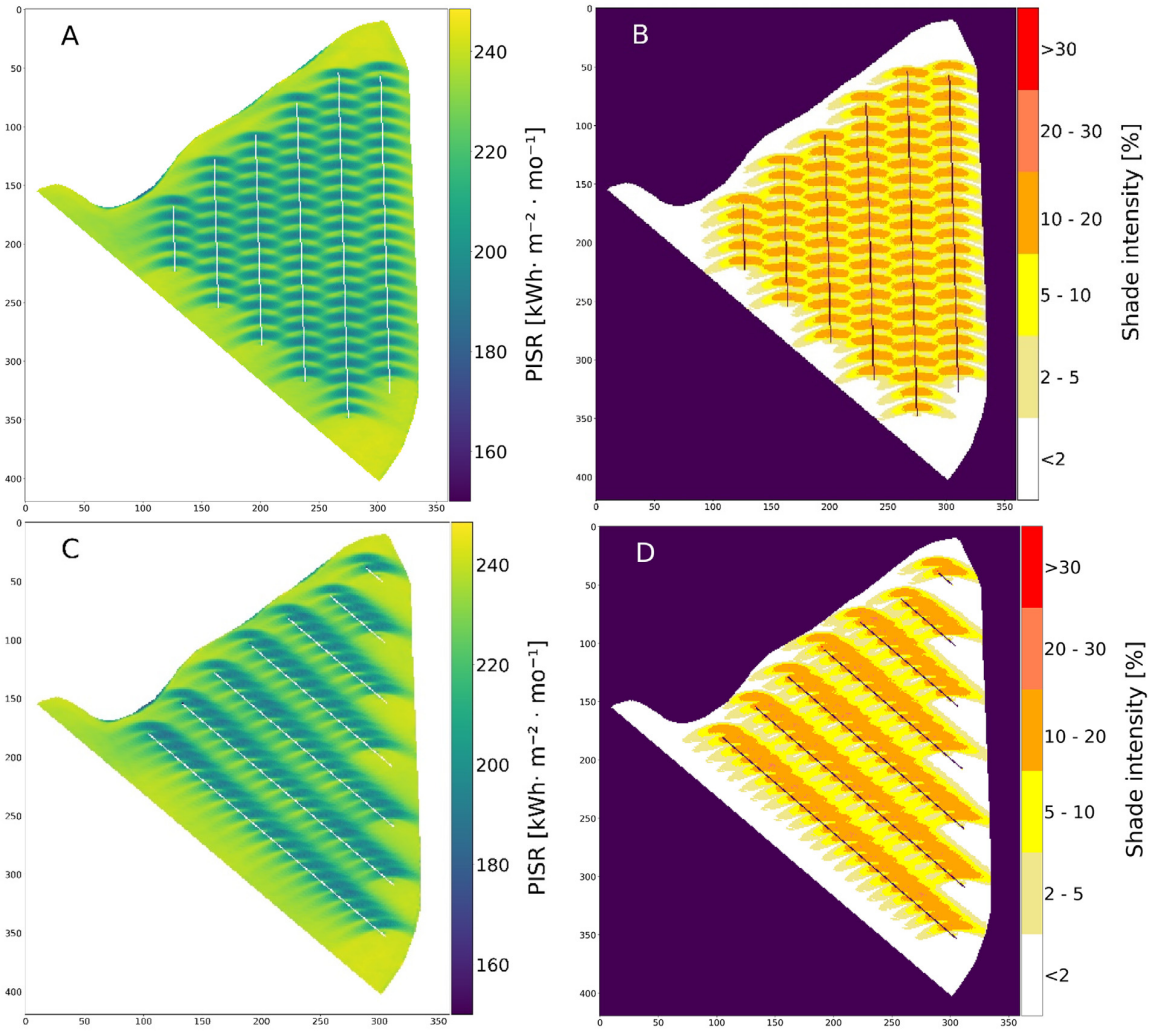
PISR and shade intensity of two different scenarios for the month of May in the year 60. A) PISR of north-south orientation B) North-south orientation and six shade intensity classes C) PISR of west-east orientation D) West-east orientation and six shade intensity classes.

There are differences in the shadow intensities and the percentage of affected areas between the two scenarios (Table 1 and Fig. 9B and D). However, there is no area of the field in either scenario that can show more than 30% PISR reduction over the course of a month. Also, the areas of the field with PISR reductions of 20% to 30% are negligible. The PISR reductions amounted 10% to 20% between the scenarios in September (Table 1). In the north-south scenario, more than half of the total field was shaded, whereas in the west-east scenario only 39.7% was shaded.

**Table 1:** PISR reduction in six shade intensity classes for both scenarios in year 60 and the months of May, July and September and the indication of total affected areas in the field.

Shade intensity	North-South Scenario			West-East Scenario		
	May	July	September	May	July	September
< 2%	25.3	27.0	18.9	30.3	33.1	23.6
≥ 2% < 5%	17.6	18.3	9.9	17.2	16.4	13.1
≥ 5% < 10%	24.1	22.0	16.1	22.6	20.9	23.1
≥ 10% < 20%	32.1	31.6	52.8	29.4	28.7	39.7
≥ 20% < 30%	0.9	1.1	2.3	0.5	1.0	0.5
≥ 30%	0	0	0	0	0	0

**Fig. 10:**
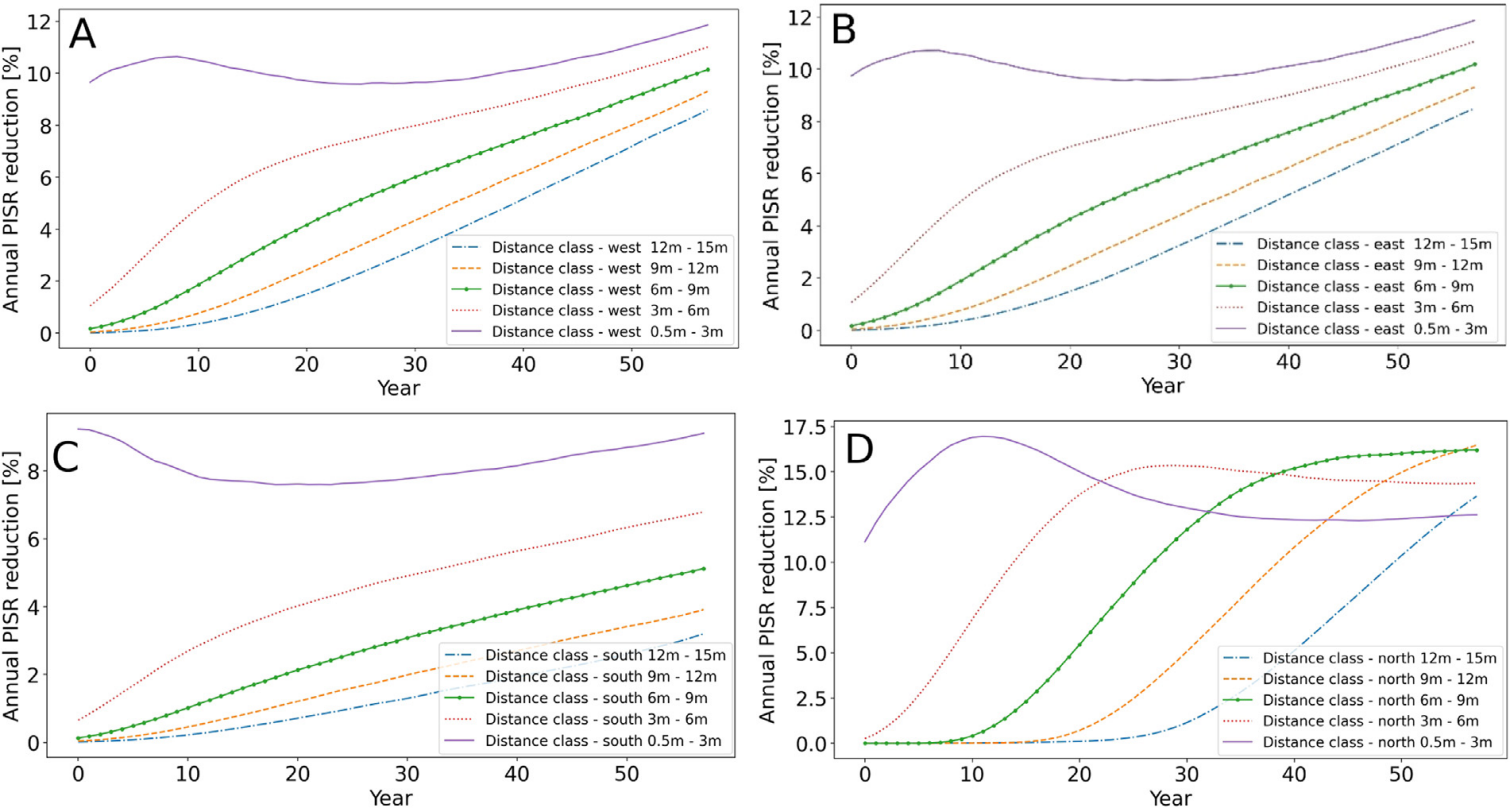
Annual PISR reduction depending on tree growth and distance to tree rows. A) North-south scenario and all distance classes west of the tree rows, B) North-south scenario and all distance classes east of the tree rows. C) West-east scenario and all distance classes south of the tree rows, D) West-east scenario and all distance classes north of the tree rows.

Of particular interest is the temporal development of PISR reduction as a function of tree growth and distance to the tree strips. The western and eastern distance classes of the north-south scenario showed minimal differences in their PISR reduction (Fig. 10A and B). This can be explained by the almost balanced shading of the western and eastern side. The distance class 0.5 to 3 m, which is very close to the tree rows, starts with high annual PISR reductions, but do not increase as much over time as the other distance classes. The most distant distance classes are also increasingly shaded over time by larger trees and larger crowns. After 60 years of tree growth, the eastern and western distance classes 12 to 15 m of the north-south scenario have 8.5% and 8.6% annual PISR reduction, respectively (Fig. 10A and B).

The PISR reduction in the west-east scenario differs in pattern and intensity from the north-south scenario. The southern distance class 0.5 to 3 m closest to the tree row starts with an annual PISR reduction of 9.2% and has a PIRS reduction of 9.1% after 60 years (Fig. 10C). In comparison, the 0.5 to 3 m distance class on the northern side of the west-east scenario starts with an annual PISR reduction of 11.1%, increases steeply in the first years to a maximum of 17.0% in the 12th year, and then decreases again to 12.6% in year 60 (Fig. 10D). The remaining distance classes on the northern side of the west-east scenario are shaded more intensively than the 0.5 to 3 m distance class in year 60. This relatively low PISR reduction of the 0.5 to 3 m distance class after 60 years is due to the fact that after this long time period the trees have reached a certain size and shade nearby areas on the northern side less strongly.

## Conclusion and future work

The design of alley cropping systems and the spatial arrangement of tree rows is complex and takes into consideration various trade-offs such as field geometry, main wind directions or terrain slopes. North-south orientated tree rows are recommended for temperate agroforestry to minimize shading of agricultural used areas. If a north-south orientation is not possible or not desired to provide extra shade as a possible adaptation strategy to climate change, simple decision support systems are not yet available. The shade simulation tool ShadOT was presented, which relies on a limited number of inputs. By simulating tree growth over several decades, potential shaded areas can be identified and compared between scenarios and sole cropped fields. Varying spatial arrangements of tree rows or single trees lead to different spatial–temporal shading patterns which can be considered in decision processes. However, only simple parameters are used and linear growth is assumed without considering site characteristics, management and climate. Future efforts should aim to incorporate these mentioned points in order to further improve the tool.

## CRediT authorship contribution statement

**Marco Donat:** Conceptualization, Methodology, Software, Writing – original draft, Visualization. **Jonas Geistert:** Methodology, Software, Writing – review & editing. **Kathrin Grahmann:** Writing – review & editing, Supervision. **Sonoko D. Bellingrath-Kimura:** Writing – review & editing, Funding acquisition, Supervision.

## Declaration of Competing Interest

The authors declare that they have no known competing financial interests or personal relationships that could have appeared to influence the work reported in this paper.

## Data Availability

Data will be made available on request.
